# Medical student intentions to practice internal medicine in underserved areas associated with debt, identity and extracurricular participation

**DOI:** 10.1186/s12909-023-04392-0

**Published:** 2023-06-08

**Authors:** Aaron Lapidus, Sapan Shah, Meheret Mekonnen, Joseph Araj, Mytien Nguyen, Hyacinth Mason, Branden Eggan, Inginia Genao

**Affiliations:** 1 Department of Medical Education and Community Outreach, Albany Medical College, Albany, NY USA; 2 MD-PhD Program, Yale School of Medicine, New Haven, CT USA; 3 Tufts University School of Medicine, Boston, MA USA; 4 Department of Nursing, Siena College, Loudonville, NY USA; 5 Office of Diversity, Equity and Belonging, Penn State College of Medicine, 700 HMC Crescent Road, Hershey, PA 17033 USA

**Keywords:** Underserved, Career, Debt, Identity, Extracurriculars

## Abstract

**Background:**

Currently, Internal Medicine (IM) physicians do not reflect the ethno-racial diversity of the US population. Moreover, there is a shortage of IM physicians in Medically Underserved Areas (MUAs) in the US. The purpose of this study was to determine factors that influence medical students’ intent to practice IM in MUAs. We hypothesized students with intentions to pursue a career in IM and work in MUAs were more likely than their peers to identify as underrepresented in medicine (URiM), report greater student debt loads, and report medical school experiences in cultural competencies.

**Methods:**

We analyzed de-identified data of 67,050 graduating allopathic medical students who completed the Association of American Medical Colleges’ (AAMC) Medical School annual Graduation Questionnaire (GQ) between 2012–2017 by multivariate logistic regression models, examining intent to practice IM in MUAs based on respondent characteristics.

**Results:**

Of 8,363 students indicating an intent to pursue IM, 1,969 (23.54%) students also expressed an intent to practice in MUAs. Students awarded scholarships, (aOR: 1.23, [1.03–1.46]), with debt greater than $300,000 (aOR: 1.54, [1.21–1.95], and self-identified non-Hispanic Black/African American (aOR: 3.79 [2.95–4.87]) or Hispanic (aOR: 2.53, [2.05–3.11]) students were more likely than non-Hispanic White students to indicate intent to practice in MUAs. This pattern also existed for students who participated in a community-based research project (aOR: 1.55, [1.19–2.01]), had experiences related to health disparities (aOR: 2.13, [1.44–3.15]), or had experiences related to global health (aOR: 1.75, [1.34–2.28]).

**Conclusions:**

We identified experiences and characteristics that associate with intention to practice IM in MUAs, which can aid future curricular redesign by medical schools to expand and deepen comprehension of health disparities, access to community-based research, and global health experiences. Loan forgiveness programs and other initiatives to increase recruitment and retention of future physicians should also be developed.

## Background

It is well documented that there is a shortage of physicians in the US, which the Association of American Medical Colleges (AAMC) predicts will reach up to 90,000 by 2025 [1]. This shortage is especially being felt in the field of Internal Medicine (IM), which plays a critical role in providing complex and comprehensive care to the aging adult population [2, 3]. Among both IM subspecialties and general IM/primary care physicians, the AAMC estimates a shortage of 3,800—13,400 physicians and 21,400—55,200 physicians by 2033, respectively [4]. For the purposes of this research, we define general IM physicians as those physicians who primarily practice in-patient care in the hospital setting who have completed training in internal medicine residency, focused on the comprehensive care of adults, but choose not to subspecialize to focus on any one organ system or problem, and primary care IM physicians as those physicians who primarily practice in the outpatient or ambulatory setting who have completed training in IM residency but choose not to subspecialize. It is important to note many physicians take on both general IM hospitalist and IM primary care roles [5].

This shortage is predicted to be most pervasive in Medically Underserved Areas (MUAs) [6], characterized as such by the Federal Government due to a combination of low primary care access, elevated infant mortality rate, elevated family poverty rate, and elevated population above age 65 [7], widening existing disparities in access to care in the U.S. [8, 9].

It is also widely recognized that the majority of actively practicing IM physicians are white (68.4% in 2018), and do not reflect the ethno-racial diversity of the US population. Disparities in representation of physicians practicing in medically underserved communities are wider as these populations have greater healthcare needs [10]. Students identifying as underrepresented in medicine (URiM)- defined by the AAMC as “racial and ethnic populations underrepresented in the medical profession relative to their numbers in the general population” [11]- are more likely than non-URiM students to plan to work with medically underserved communities [12]. Furthermore, in 2018, 14.4% of practicing general and primary care internists were URiM, compared with 12.9% of practicing physicians among all specialties [13].

Among 2010–2012 AAMC GQ respondents, 54.8% of students identifying as URiM indicated an intention to work with underserved populations, compared to 29.1% of their non-URiM counterparts [14]. However, the challenge of low URiM representation among medical school matriculants persists. Notably, only 18.1% of matriculants to medical school in 2018 identified as URiM, substantially less than the 31.5% of individuals within the US population characterized as URiM [15]; therefore, it follows that increasing URiM matriculants may lessen the gap of IM physicians aiming to serve in MUAs. The intent to work in MUAs in the field of IM may be linked to student debt load as well; URiM students accrue, on average, more student debt as compared to their non-URiM counterparts [16].

While IM provides some opportunities to work with underserved communities, there is limited availability to do so during IM residency as compared to other specialties (such as family medicine or pediatrics) [12, 14]. This may play a role in influencing physician demographics within IM as a specialty.

Since the diverse factors that influence a medical student’s decision to practice IM in MUAs have not been well-investigated, we determined to examine both demographics and characteristics that are associated with this intent in our study. Based on the outlined links observed in prior studies examining practice in other specialties, we initially hypothesized that students demonstrating intent to pursue a career in IM and work with underserved communities were more likely to identify as URiM, more likely to report high debt loads, and more likely to report medical school experiences in cultural competencies.

## Methods

De-identified, individual-level data was obtained from the AAMC Student Record System (SRS) for 67,050 U.S. allopathic medical school matriculants from the periods of 2007–2008 and 2012–2017 who completed the AAMC Graduation Questionnaire (GQ), a national survey of graduating medical students that is administered annually between February and June [17] The study’s data include the following data from the AAMC SRS and GQ: demographic data including age at matriculation, self-reported ethno-racial identity and sex, degrees obtained upon graduation, financial data including total debt upon graduation and self-reported scholarship attainment, and curricular data including self-reported participation in various electives during medical school based on recall. For electives, numerous options were available, for example self-reported participation in “global health experiences” (with no further breakdown of specific activities within this field), providing “health education” (i.e. HIV/AIDS education, breast cancer awareness etc.) or “experience with a free clinic for the underserved population”. Additional data included intended practice specialty, and intention to practice in an underserved area. Age at matriculation was categorized as a binary variable to identify students who were aged 23 years or older when entering medical school. These individuals are typically considered ‘non-traditional’ matriculants who gain experience in the workforce prior to entering medical school. Self-reported ethno-racial identity was grouped as: Hispanic, non-Hispanic (NH) White, NH Black/African American, NH Asian, NH American Indian or Alaska Native, NH Hawaiian Native or Other Pacific Islander, and Unknown/Other. Students who reported identifying with more than one ethno-racial identity were classified as Multiracial. Total student debt at graduation was categorized into 5 levels: no debt; debt less than $100,000, debt from $100,000–$199,999, debt from $200,000-$299,999, or debt greater than or equal to $300,000. Students who reported an intention to pursue IM, whether that be general hospital internal medicine, primary care internal medicine, or an internal medicine sub-specialty, were classified as “Internal Medicine.” Students’ intention to practice in MUAs are considered to be “yes” if a student indicated “yes”, and “no” if the student indicated “undecided” or “no.”

All data collected for this study were de-identified, and only authorized users were allowed access to the data. This study was deemed exempt by the Albany Medical College Institutional Review Board. Students who did not complete the demographics section of the GSQ or who did not indicate an intention for a specific medical specialty were excluded from the analysis. Frequencies and percentages were utilized for descriptive statistics for demographic factors associated with intention to pursue IM. Then, Chi-squared testing was performed to determine significance in distribution between percentage of students reporting intention to pursue IM versus other specialties. We analyzed the data via multivariate logistic regressions model and calculated adjusted odds ratios (aOR) for the effects of covariates on students’ intention to pursue IM and practice in MUAs. This multivariate model enabled us to look for effects of covariates on intention to practice IM in MUAs accounting for potential confounders. All aORs were reported with 95% confidence intervals. All statistical analyses were performed using *STATA* 16.1.

## Results

### Characteristics of students intending to pursue internal medicine

A total of 67,050 medical students in their final year of medical school completed the AAMC GQ from 2012–2017, including the question about career preference. From the respondents, 18,948 were excluded from the study due to missing key demographic and financial information, leaving a total of 48,102 students in the study. Of this population, 8,363 (17.38%) reported an intention to pursue IM.

Table 1 characterizes respondents reporting interest in IM versus students reporting interest in non-IM specialties. Considering race and ethnicity, a lower proportion of students interested in IM identified as URiM (15.88% vs. 16.53%; χ^2^ *p* < 0.001). A higher proportion of students interested in IM also reported accruing no debt (20.08% vs. 15.05%; χ^2^ *p* < 0.001) or debt less than $100,000 (18.34% vs. 16.72% χ^2^ *p* < 0.001). However, a lower proportion reported debt between $100,000-$199,999 (29.58% vs. 31.34% χ^2^ *p* < 0.001), $200,000-$299,999 (24.70% vs. 38.32% χ^2^ *p* < 0.001) or greater than $300,000 (7.30% vs. 8.57% χ^2^ *p* < 0.001) compared to students interested in other specialties (χ^2^ *p* < 0.001). Additionally, a lower proportion of non-IM students reported acquiring a scholarship towards the costs of medical school (58.20% vs. 61.65%, χ^2^ *p* < 0.001).

**Table 1:** Graduating Medical Students from U.S. Allopathic Medical Schools in Matriculation Years 2007–2008 through 2011–2012

Characteristic (N (%))	Non-IM Students (*N* = 39,739)	IM Students (*N* = 8,363)
Race/Ethnicity***		
NH White	26,681 (67.14)	4,831 (57.77)
Aggregate URiM	6,570 (16.53)	1,328 (15.88)
NH Asian	6,488 (16.33)	2,204 (26.35)
NH Black/African-American	1,669 (4.20)	355 (4.24)
NH Native American/Alaska Native	78 (0.20)	12 (0.14)
Hispanic	2,732 (6.87)	506 (6.05)
NH Hawaiian Native/Other Pacific Islander	63 (0.16)	10 (0.12)
NH Multiracial	1,117 (2.81)	227 (2.71)
NH Unknown/Other	911 (2.29)	218 (2.61)
Total Debt***		
No debt	5,226 (15.05)	1,476 (20.08)
< 100,000	5,809 (16.72)	1,348 (18.34)
$100,000–$199,999	10,885 (31.34)	2,175 (29.58)
$200,000-$299,999	9,838 (28.32)	1,816 (24.70)
> $300,000	2,977 (8.57)	537 (7.30)
Intention to Practice in Underserved Areas***		
Yes	10,565 (26.71)	1,969 (23.67)
Acquired a scholarship***		
Yes	24,388 (61.65)	4,840 (58.20)
Experience in providing health education in the community***		
Yes	15,981 (40.21)	3,154 (37.71)
Participated in a community-based research project*		
Yes	10,905 (27.44)	2,185 (26.13)
Experience related to cultural awareness and cultural competency**		
Yes	27,531 (69.28)	5,669 (67.79)
**Participated in educating students about careers in health professions or biological sciences**		
Yes	18,350 (46.18)	3,441 (41.24)
**Experience with a free clinic for the underserved population**		
Yes	29,455 (74.12)	6,148 (73.51)
**Experience related to health disparities**		
Yes	27,374(68.88)	5,702 (68.18)
**Global health experience**		
Yes	12,085 (30.41)	2,478 (29.63)
**Learned the proper use of the interpreter when needed**		
Yes	29,638 (74.58)	6,174 (73.83)
**Learned another language to improve communication with patients**		
Yes	9,739 (24.51)	1,973 (23.59)

*Abbreviations*: *NH* Non-Hispanic, *SE* Standard Error, *CI* 95% Confidence Interval

Significance: * *p* < 0.05; ** *p* < 0.01; *** *p* < 0.001

Students interested in IM were less likely to report an intention to practice in MUAs (23.67% vs. 27.61%, χ^2^ *p* < 0.001) than those interested in a non-IM specialty. They also were less likely to report experience ‘providing community-based health education’ (37.71 vs. 40.21%, χ^2^ *p* < 0.001), ‘participating in a community-based research project’ (26.13% vs 27.44%, χ^2^ *p* < 0.05), or ‘participating in educating students about careers in health professions and biological sciences’ (41.24% vs 46.18%, χ^2^ *p* < 0.001). Furthermore, students interested in IM were less likely to report ‘experience related to cultural awareness and cultural competency’ (67.79% vs. 69.28%, χ^2^ *p* < 0.05). All other characteristics and experiences of medical students interested in IM versus other specialties were very similar (within 1%) of one another.

### Intention to practice in underserved areas

Of all respondents reporting interest in IM, 1,969 (23.67%) reported an intention to practice in MUAs. In contrast, 6,394 (76.46%) reported no intention to practice in these areas. Table 2 reports the characteristics of respondents interested in practicing IM in MUAs. The following variables were associated with intention to practice in MUAs: attainment of a scholarship (aOR: 1.26 [1.12–1.42]), debt load ranging from $100,000-$199,999 (aOR: 1.30, [1.09–1.55]) and greater than $300,000 (OR: 1.54, [1.21–1.95]), identification as NH Black/African-American (aOR: 3.79 [2.95–4.87]), Hispanic (aOR: 2.53, [2.05–3.11]), or NH Unknown/Other (aOR: 1.61, [1.16–2.23]), participation in a community-based research project (aOR: 1.55, [1.19–2.01]), experience related to health disparities (aOR: 2.13, [1.44–3.15]), and global health experience (aOR: 1.75, [1.34–2.28]).

**Table 2:** Graduating Allopathic Medical Students’ Intention to Practice in Underserved Areas- Multivariate Logistic Regression Analysis

Covariate or Predictor (Reference)	Adjusted Odds Ratio (95% CI)
Age at matriculation greater than or equal to 23 years old***	0.68 (0.61–0.77)
Scholarship (No scholarship)***	1.26 (1.12–1.42)
Total Debt at Graduation (No debt)	
< 100,000	1.06 (0.87–1.29)
$100,000–$199,999**	1.30 (1.09–1.55)
$200,000-$299,999	1.20 (1.00–1.44)
> $300,000***	1.54 (1.21–1.95)
Ethno-racial groups (NH White)	
NH Asian	0.96 (0.84–1.11)
NH Black/African-American***	3.79 (2.95–4.87)
NH Native American/Alaska Native	1.45 (0.42–4.95)
Hispanic***	2.53 (2.05–3.11)
NH Native/Hawaiian Native/Other Pacific Islander	0.43 (0.053–3.53)
NH Multiracial	1.23 (0.88–1.72)
NH Unknown/Other**	1.61 (1.16–2.23)
Experience in providing health education in the community	1.17 (0.91–1.50)
Participated in a community-based research project**	1.55 (1.19–2.01)
Experience with a free clinic for the underserved population	1.14 (0.83–1.57)
Experience related to health disparities***	2.13 (1.44–3.15)
Global health experience***	1.75 (1.34–2.28)
Learned the proper use of the interpreter when needed*	0.71 (0.51–0.98)
Learned another language to improve communication with patients	1.22 (0.92–1.61)

*Abbreviations: NH* Non-Hispanic, *SE* Standard Error, *CI* 95% Confidence Interval

Significance: * *p* < 0.05; ** *p* < 0.01; *** *p* < 0.001

## Discussion

We believe that this is the first national study focused on investigating potential factors influencing medical student intention to specifically pursue IM and work in MUAs. Our study had three major findings related to students interested in IM who also expressed an intention to practice in MUAs. These students were: 1) more likely to identify as NH Asian, NH Black/African American, or NH Unknown/Other race, 2) more likely to report extracurricular experiences centered on community-based research, health disparities, or global health, and 3) more likely to report greater debt loads ($100,000-$199,999 or > $300,000) and/or attainment of a scholarship. These findings confirm our initial hypothesis, but are especially striking when compared with some of our other findings, which showed students interested in IM *overall* were: 1) more likely to report lower debt loads, 2) less likely to identify as URiM, 3) less likely to report the above-noted extracurricular experiences, and 4) less likely to report interest in working in MUAs (as compared to the overall graduating medical student population).

Conducting this research study enabled us to identify experiences and characteristics that correlate with intent to practice IM in MUAs, and reinforced existing evidence examining characteristics of trainees pursuing practice in rural and urban MUAs. Based on the findings of our quantitative study, we call for specific actions by medical school leadership to promote capacity among their school’s students to alleviate the ever-growing gaps in health care delivery in MUAs. Furthermore, we believe that our study findings can aid decisions promoting curricular redesign by medical schools to foster increased comprehension of health disparities, access to community-based research, and global health experiences. The current state of healthcare access disparities in the US combined with our study’s analysis results suggests strongly that undergraduate institutions and medical schools should actively promote specific extracurricular opportunities to work in community settings and MUAs; solidifying existing evidence on a smaller scale indicating participation in MUAs as early as high school and as late as residency training is an independent predictor of intention to practice in MUAs [1, 18, 19].

Additionally, programs can look to previous medical training practices successfully implemented to increase the proportion of graduates who practice in medically underserved areas. One meta-analysis examining 130 studies of interventions found that attending a medical school or experiencing postgraduate training in an underserved area were positively associated with eventual practice in said areas [20]. Maximizing exposure to medical practice in MUAs early in the medical school experience by offering, promoting, and/or requiring community or global health volunteering and involvement among students could be a potent solution to address physician shortages in IM MUAs [19, 21].

Our findings support the notion that a more diverse medical school student body may result in more physicians practicing in MUAs. It is well understood that URiM physicians are more likely to want to serve members of their own ethno-racial population group, even after accounting for socioeconomic differences of the practice setting [1, 19]. By supporting URiM students through these interventions, medical institutions can play a tangible role in creating a more representative physician workforce better equipped to specifically address gaps in MUAs, consisting of patients in most need of healthcare.

We can also look to other medical specialties for successful measures implemented to recruit and retain a diverse physician population and practice in MUAs. One study examined the implementation of a four-year extracurricular experience during medical school that included dedicated clerkship, mentoring, and scholarly projects focused on underserved populations and found a statistically significantly higher proportion of those who graduated from the program matched into Family Medicine as compared to non-participants [22]. In the field of Emergency Medicine, “The Council of Emergency Residency Directors” (CORD) outlined 7 diverse recruitment practices which included recognition of applicant/faculty diversity in the interview process, development of curriculum to address topics of diversity, implicit bias and cultural competence. Residency programs that have implemented a minimum of 2 of these CORD practices have both more diverse faculty and residents [23].

In addition, addressing disparities in student debt (and debt influence on practice choice) could also promote increased student intent to practice IM in MUAs. Currently, non-military loan assistance programs in the US are centered around *Public Service Loan Forgiveness (PSLF)*. The *PSLF* Program forgives the remainder of participant student loans following 10 years of qualifying payments, as long as the physician in any specialty remains employed at a not-for-profit institution during their loan payback period, and the *National Health Service Corps (NHSC)*, which provides a limited number of applicants up to $50,000 in payments towards student loans for physicians who elect to practice primary care (General IM, Obstetrics and Gynecology, Family Medicine, or Psychiatry) for at least 2 years in eligible MUAs, and has been shown to provide high-value care [24, 25]. While participation and interest in the *PSLF* program is clearly present (62% of 2014 AAMC GQ respondents seeking loan forgiveness reported interest in this program), this is not the case for the more targeted *NHSC* program, as future physicians intend to use *PSLF* more than the *NHSC* [26]. Problematically, many students who would be inclined to practice Internal Medicine in MUAs with financial assistance are deterred by the often complex and narrow eligibility requirements of these programs. In turn, this leaves these students at a financial disadvantage as compared to those practicing care outside of MUAs [14, 27]. Modifying these programs to be both better promoted and simpler to access for students choosing to practice Internal Medicine and other specialties in MUAs would be a sensible approach to address shortages in these areas. It is crucial to focus on URiMs who indicate an interest in practicing in MUAs when restructuring loan repayment programs. Our study shows that students with higher debts were more likely to express intent to practice in MUAs. Studies also demonstrate that URiM have a disproportional medical education debt burden [27]. These two factors combined are drivers for action to decrease education debt, particularly for those serving MUAs.

## Limitations

This study was limited by its reliance on collected survey data focused on intention to practice in the field of IM and in MUAs. We did not have access to the data that would have enabled us to link to actual match in, or practice of IM. At the same time, previous research examining the validity of graduating students who indicate intention to practice a specific specialty are more likely to fulfill these intentions and practice in MUAs during residency and as attending physicians, particularly in primary care [28–30], and a single-center study found similar conclusions regarding intention to practice in MUAs [31]. Similarly, this study considered participation in extracurricular experiences based on student recall, which may have been affected by recall bias; for example, curricular experiences offered which were not meaningful to students and thus not recalled when answering the GQ may have been underreported. Additionally, this study did not consider the characteristics of international medical graduates, who play a major role in helping address physician shortages in MUAs [32], nor did the sample include osteopathic medical school students- it was administered solely to allopathic medical school students. Due to a smaller sample size, the category of ethnic “Asian” was not further subdivided. Furthermore, this study does not differentiate internal medicine by practice in subspecialties, hospitalist medicine, or primary care internal medicine, and does not differentiate intention to practice in rural MUAs versus urban MUAs. Future studies are needed to assess long-term how medical student intention to practice in MUAs translates to practice setting after residency training. Further studies clarifying the characteristics of IM residents intending on pursuing IM subspecialties could also be beneficial considering the variety within the field of IM.

## Conclusion

Students interested in IM indicating intention to practice in MUAs were more likely to identify as URiM, had specific extracurricular experiences related to working in underserved areas, and reported higher debt loads than those who did not express this interest. To fill existing gaps in IM care, which are especially felt in MUAs, medical education institutions at both the undergraduate and postgraduate levels of training can place admission and recruitment policies facilitating a more diverse medical student body, increased opportunities for experience with global health, community research, and/or health disparities and increased awareness about programs that ease the debt load of future physicians choosing to practice in MUAs. Wide implementation of these measures can serve as a starting point to fill much needed gaps in culturally competent care among the aging US adult population.

## Data Availability

The dataset analyzed during the current study are property of the Association of American Medical Colleges and are not publicly accessible but are available from the corresponding author on reasonable request.
